# Identification of genes involved in low aminoglycoside-induced SOS response in *Vibrio cholerae*: a role for transcription stalling and Mfd helicase

**DOI:** 10.1093/nar/gkt1259

**Published:** 2013-12-05

**Authors:** Zeynep Baharoglu, Anamaria Babosan, Didier Mazel

**Affiliations:** ^1^Département Génomes et Génétique, Unité Plasticité du Génome Bactérien, Institut Pasteur, 75015 Paris, France and ^2^Centre National de la Recherche Scientifique, CNRS, UMR3525 Paris, France

## Abstract

Sub-inhibitory concentrations (sub-MIC) of antibiotics play a very important role in selection and development of resistances. Unlike *Escherichia coli*, *Vibrio cholerae* induces its SOS response in presence of sub-MIC aminoglycosides. A role for oxidized guanine residues was observed, but the mechanisms of this induction remained unclear. To select for *V. cholerae* mutants that do not induce low aminoglycoside-mediated SOS induction, we developed a genetic screen that renders induction of SOS lethal. We identified genes involved in this pathway using two strategies, inactivation by transposition and gene overexpression. Interestingly, we obtained mutants inactivated for the expression of proteins known to destabilize the RNA polymerase complex. Reconstruction of the corresponding mutants confirmed their specific involvement in induction of SOS by low aminoglycoside concentrations. We propose that DNA lesions formed on aminoglycoside treatment are repaired through the formation of single-stranded DNA intermediates, inducing SOS. Inactivation of functions that dislodge RNA polymerase leads to prolonged stalling on these lesions, which hampers SOS induction and repair and reduces viability under antibiotic stress. The importance of these mechanisms is illustrated by a reduction of aminoglycoside sub-MIC. Our results point to a central role for transcription blocking at DNA lesions in SOS induction, so far underestimated.

## INTRODUCTION

*Vibrio cholerae* is a human pathogen that grows planktonically or as biofilms on crustacean shells, where it couples genome plasticity and adaptation to a changing environment, through the modulation of the SOS response. SOS induction takes place when single-stranded DNA (ssDNA) is detected in the cell and activates recombination and repair pathways, which results in increased mutagenesis and genome rearrangements (1). Induction of the SOS response also leads to cassette rearrangements in the *V. cholerae* superintegron, ultimately allowing the expression of adaptation factors, like antibiotic resistance genes (2). It is now established that horizontal gene transfer, which involves the uptake of ssDNA by bacteria, also induces the SOS response (2,3).

The SOS response is also induced upon DNA damage, for instance, after ultraviolet (UV) irradiation or treatment with antibiotics that target DNA replication, such as fluoroquinolones. We previously studied the effect of sub-minimal inhibitory concentrations (sub-MICs) of antibiotics from different structural families on induction of the SOS response in *V. cholerae* and *Escherichia coli.* Sub-MICs of antibiotics are commonly found in various environments due to the increased production and use of antibiotics for the treatment of humans and animals. Their presence in the environment (4–7) can be a potent stressor for bacteria and likely plays a very important role for selection of resistances (8). We observed that sub-MICs of aminoglycosides (AGs) induce the SOS response in *V. cholerae* but not in *E. coli* (9). This observation was interesting, as AGs do not target DNA but rather affect translation. We obtained evidence showing that low AG concentrations actually induce the incorporation of oxidized guanine (8-oxo-G) residues from the dNTP pool in *V. cholerae*, but not in *E. coli*, and that this plays a role in the formation of ssDNA intermediates in *V. cholerae* (thus induction of SOS) (10). We also observed that the stress sigma factor RpoS has a protective role against induction of the SOS response by sub-MIC tobramycin (TOB) in *E. coli* and *V. cholerae* (10). We decided to try to shed light onto the mechanisms of ssDNA formation after 8-oxo-G incorporation in DNA.

The 8-oxo-G lesions are known to be mutagenic for both DNA and RNA polymerases (RNAP) (11): the 8-oxo-G residue is paired with an adenine instead of a cytosine by the DNA polymerase. *E. coli* and *V. cholerae* have a defense system against 8-oxo-G incorporation and incorrect pairing, which involves the MutT, MutY and MutM proteins (12). MutT hydrolyses 8-oxo-G in the nucleotide pool (13). *mutT* inactivation causes mistranscription and affects the fidelity of DNA replication (14). MutY and MutM belong to the base excision repair (BER) pathway (15): inactivation of *mutM* leads to the accumulation of 8-oxo-G in cells, which results in GC to TA transversions (16). MutY recognizes and excises the adenine of the G–A mispair (17). Incomplete action of the BER system may lead to double-stranded DNA (dsDNA) breaks that are cytotoxic if unrepaired (18). The BER pathway was shown to be important in the response to sub-MIC AG treatment in *E. coli* because a BER mutant strain induced the SOS response in the presence of TOB, whereas the wild-type strain did not (10). The mismatch repair pathway (MMR) is also involved in DNA mismatch correction. Unlike for BER, MMR does not repair lesions but rather repairs mismatches that are formed during replication: MutL and MutS proteins recognize the mismatch, MutH nicks the unmethylated (i.e. new) DNA that is removed by UvrD and the gap is filled by resynthesis. It was shown that MMR provides additional protection to BER against 8-oxo-G-mediated mismatches (19–21).

The induction of SOS after AG treatment implies that the formation of ssDNA intermediates is associated with the incorporation of 8-oxo-G. R-loops are one type of structure that can be formed at incorporated 8-oxo-G, and R-loop formation can lead to SOS induction through dsDNA break formation (22,23). R-loops are RNA–DNA hybrids that are formed when the RNAP is stalled on DNA and the RNA molecule in synthesis anneals to its homologous ssDNA template in *E. coli* [reviewed in (24)] and eukaryotes (25). RNAP back-tracking may also promote the formation of DNA–RNA hybrids (26). R-loops were shown to lead to genomic instability in *E. coli*, as well as in eukaryotes (22), by impairing DNA replication. Even though RNAP commonly stalls at bulky DNA lesions (27), RNAP stalling and backtracking were also observed at non-bulky 8-oxo-G lesions (28,29). Furthermore, the stalled RNAP is an obstacle to replication fork progression and is dislodged by specialized proteins (24).

Several helicases are involved in the removal of the stalled RNAP, such as Mfd, which associates transcription stalling to nucleotide excision repair (NER) through a mechanism called transcription-coupled repair (TCR). In this context, Mfd recognizes and binds the RNAP stalled at a site of DNA damage and releases the RNA transcript and the RNAP enzyme from the DNA (30–32) to recruit the NER machinery (33,34). The UvrAB complex of the NER pathway recognizes the lesion and unwinds the DNA (35). UvrC makes single-strand nicks to help UvrD remove the damaged DNA region. The missing DNA is then resynthesized through gap filling.

The Mfd helicase mentioned above thus removes the RNAP complex itself to initiate repair. Other helicases, such as DinG (36) or Rep (37), are also involved in the removal of bulky protein complexes from DNA, including the RNAP stalled on DNA lesions. In this respect, DinG and Rep helicases have similar roles, and it has been observed that in the absence of Rep, DinG is essential for viability in conditions that cause RNAP to stall (36). DinG and Rep promote fork movement through transcription complexes, and DinG was proposed to inhibit the formation and accumulation of R-loops *in vivo* (38,39). The RNaseH protein, an R-loop-specific helicase, removes the R-loop and destabilizes the stalled RNAP (40). In the absence of stalled RNAP removal systems, DNA replication is impaired (24,38).

To gain further insight into mechanisms connecting AG treatment and the SOS response in *V. cholerae*, we developed a genetic screen to select for *V. cholerae* mutants that do not induce the SOS response upon exposure to sub-MIC AGs (here tobramycin) but that are still capable of inducing it in other conditions (e.g. after mitomycin C treatment leading to direct DNA damage). We found several mutants among which were genes involved in stalled RNAP removal mechanisms, pointing to an important role of 8-oxo-G lesions in impeding transcription, leading to the formation of R-loops and ultimately to SOS response induction, during repair.

## MATERIALS AND METHODS

### Strain constructions

Gene deletions were performed using the three-step polymerase chain reaction (PCR) assembly technique: oligonucleotide couples *Forward* and *Reverse* (Supplementary Table S1) were used to amplify the 500-bp regions upstream and downstream the gene of interest on the *V. cholerae* N16961 chromosome. In addition, *aadA1*, conferring resistance to spectinomycin, was amplified from the pAM34 plasmid with sequences homologous to the regions flanking the gene of interest. The three PCR products were assembled as ‘Forward region-*aadA1*-Reverse region’ using oligonucleotides indicated in bold. The assembled DNA fragment was introduced into *V. cholerae* N16961 *hapR+ El tor* strain 8637 by natural transformation as described earlier (3), and the deletion mutants were selected on spectinomycin 100 µg/ml plates. *E. coli* strains (Table 3) were constructed by P1 transduction. **A849:** P1 preparation on strain JW1100-1 (KEIO collection) and transduction in MG1655. **A868:** P1 preparation on strain JW1100-1 (KEIO collection) and transduction in 519. Plasmid constructions are described in Supplementary Table S2. Oligonucleotide sequences are shown in Supplementary Table S3.

### Construction of pTOX-SOS (p9095)

(i) P*recN-lacI* was amplified from MG1655 genomic DNA using oligonucleotides 1273/1274 carrying BamHI and SpeI restriction sites and the P*recN* promoter and a ribosome binding site. The fragment was cloned in the mentioned restriction sites in plasmid pSW23T (a conjugative plasmid with conditional replication) (41), yielding plasmid pSW23T_P*recN-lacI.* (ii) P*lac-ccdA* was amplified from p3478 carrying *ccdA* (laboratory collection) using oligonucleotides 1275/1276 carrying P*lac* and a ribosome binding site. The DNA fragment was cloned in pTOPO (Stratagene), cut out by EcoRI digestion and sub-cloned in the EcoRI site of pSW23T_P*recN-lacI.* (iii) *ccdB* was cut out from pSW7848 [laboratory collection (42,43)] using XbaI and SacI and cloned in pSW23T-P*recN-lacI*_P*lac-ccd* yielding plasmid p8772. (iv) A 1154-bp homology region to *lacI-lacZ* operon was amplified using oligonucleotides 1369/1370. The fragment was digested with SpeI and cloned in the p8772 SpeI site. This final plasmid was called pTOX-SOS. It was transformed in the donor strain β3914 following a protocol already described (43), yielding strain 9095.

### Construction of the *V. cholerae*::*mariner* library

pSC189 (44) was delivered from *E. coli* strain 7257 (β2163 pSC189::spec, laboratory collection) into 8637. Conjugation was performed for 4 h on 0.45 µM filters following a protocol previously described (41). The filter was resuspended in 5 ml of Luria Broth (LB), and several Petri dishes containing 100 µg/ml spectinomycin were spread to achieve a library size of 10 000–20 000 clones. The colonies were scraped and resuspended in 2 ml of LB.

### Construction of the *V. cholerae* genomic library

The library construction was performed as previously described (45). Briefly, purified genomic DNA from *V. cholerae* N16961 was submitted to partial digestion with Sau3AI to obtain 2–8-kb fragments, which were purified by gel electrophoresis. The genomic fragment pool was then cloned into BamHI-digested pUC19.

### Conjugation of pTOX-SOS in the *V. cholerae::mariner* library and selection of clones

An overnight culture of strain 9095 was diluted 100-fold in 5 ml of LB supplemented with 0.3 mM 2,6-diaminopimelic acid. At OD 0.5, 2 ml of culture was mixed with 2 ml of *V. cholerae::mariner* library described above. Conjugation was performed for 4 h on 0.45 µM filters following a protocol previously described (41). The filter was resuspended in 5-ml LB. One hundred microliters of the suspension was added to 5-ml LB + sub-MIC TOB at 0.01 µg/ml, and incubated with shaking at 37°C for 4 h, to enrich the culture with clones that had integrated the pTOX-SOS plasmid in their chromosome and that did not induce SOS. One hundred microliters of the culture was spread on plates containing 5 µg/ml chloramphenicol to select for plasmid integration (in the *V. cholerae* chromosome *lacI-lacZ* region) and sub-MIC TOB at 0.01 µg/ml. Chloramphenicol at selective concentration (5 µg/ml) does not induce SOS in resistant strains (not shown); only sub-MIC TOB induces SOS under these conditions. Colonies that grew were streaked on fresh plates.

### Identification of the genes interrupted by the mariner transposon

The localization was performed by arbitrary primed PCR as described elsewhere (46). Randomized primer ARB6 (46) and primers MEV288 and mariner-a-bis specific to each end region of the transposon were used. The amplification conditions were as follows: 10 min at 95°C; 5 cycles of 30 s at 95°C, 30 s at 30°C, 1 min at 72°C; 30 cycles of 30 s at 95°C, 30 s at 40°C and 1 min at 72°C. The PCR product was purified using Qiagen PCR purification kit. A second round of PCR was subsequently performed after dilution of 1 μl from the purified PCR mixture into 50 μl of a fresh PCR mixture. This second amplification (30 cycles of 30 s at 95°C, 30 s at 50°C and 1 min at 72°C; 5 min at 72°C) was carried out with oligonucleotides ARB2 (specific of ARB6) (46) and MEV288 or mariner-a-bis as primers. The PCR products were purified by migration on agarose gels and sequenced using MEV288 or mariner-a-bis.

### Measurement of doubling times

Overnight cultures were diluted 100-fold in the specified medium: LB or LB + sub-MIC TOB at 0.25 µg/ml. Growth curves were performed in a TECAN Sunrise at 37°C with shaking during 8 h. The OD_600_ was measured every 5 min. The doubling time was calculated by calculating the slope of the line fitting the exponential phase and compared with wild-type.

Flow cytometry experiments were performed as described (2,9).

### Growth curves of the *dnaAts* strain and derivatives

Overnight cultures were diluted in LB to an OD of 0.005. Cells allowed to grow at 30°C for 1 h 30 min and were then shifted to 42°C (Time 0). Appropriate dilutions were plated at the indicated times; plates were counted after 48-h incubation at 30°C. For strains carrying a plasmid, carbenicillin (100 µg/ml) was added to culture and plating media to maintain the plasmid.

UV survival tests were performed as described (47). Briefly, serial dilutions of exponential cultures (OD_600_ ≈ 0.5) of different strains were plated. Plates were UV irradiated at 0/40/60 J/m^2^ and incubated for 24 h at 37°C. The ratios of the numbers of colonies on irradiated plates to those on non-irradiated plates were calculated.

## RESULTS

### Identification of genes involved in SOS induction after sub-MIC aminoglycoside treatment in *V. cholerae*

We constructed and screened a *V. cholerae* mariner transposon insertion library to search for specific factors that would allow sub-MIC of AGs to induce the SOS response.

**Figure 1.**
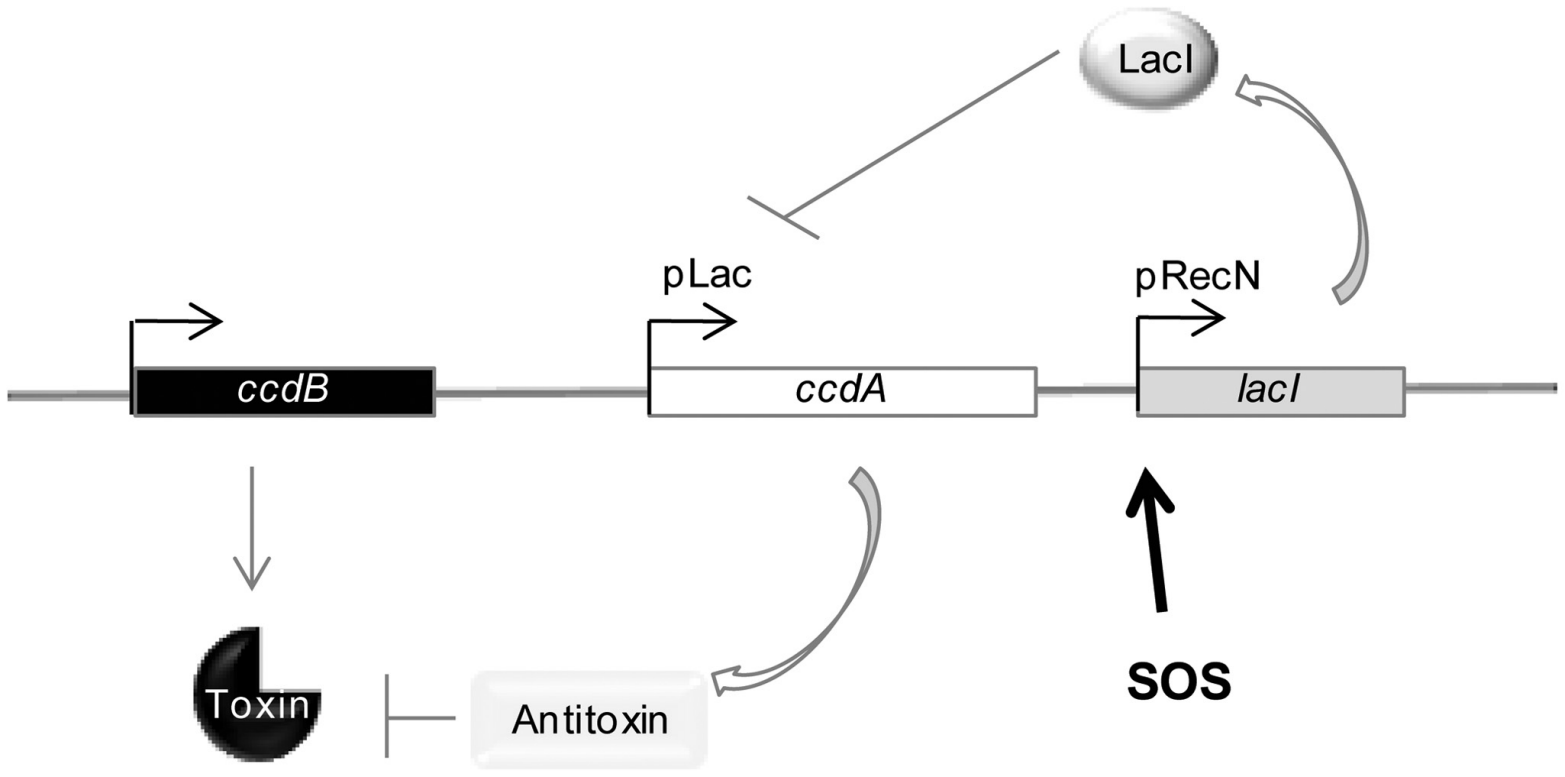
Plasmid pTOX-SOS. Described in the text. Briefly, this plasmid codes for a CcdAB toxin–antitoxin system derived from the plasmid F toxin–antitoxin module. The CcdB toxin blocks the gyrase activity and is lethal, except when bound by the CcdA antitoxin. The *ccdA* antitoxin gene is placed here under the control of a *lac* promoter that can be repressed by LacI, and the LacI production is placed under the control of the SOS-inducible *recN* promoter. When SOS is induced, *lacI* transcription from the *recN* promoter is induced. LacI represses the *lac* promoter and CcdA expression. The absence of CcdA expression results in CcdB toxicity for the cell carrying this construct. In summary, when SOS is induced, the CcdB toxin kills the bacteria, and when SOS is repressed, the CcdB toxin is inactivated. Thus, only bacteria unable to induce SOS survive to form colonies on sub-MIC antibiotics.

To do that, we designed a synthetic conditionally replicative and integrative plasmid, pTOX-SOS, that allows for the counter-selection of bacteria that induce SOS (Figure 1). This plasmid encodes the CcdAB toxin–antitoxin system derived from plasmid F, in which the CcdB toxin blocks the activity of the gyrase and is lethal except when bound by CcdA, its antidote. In our setup, the *ccdA* antitoxin gene is placed under the control of the *lac* promoter, which is repressed by LacI, itself artificially placed under the control of the SOS-inducible *recN* promoter. When SOS is induced, the transcription of *lacI* from the *recN* promoter is induced, leading to repression of CcdA expression from the *lac* promoter. The absence of CcdA expression results in CcdB toxicity and cell death. Thus, only the cells unable to induce the SOS response survive to form colonies on sub-MICs of antibiotics. pTOX-SOS also carries the R6K origin of replication, which requires the presence of the Pir protein to replicate. As this protein is not present in the genome of *V. cholerae*, the plasmid has to be integrated by homologous recombination in the chromosome to be maintained. This allows the expression of the CcdB toxin from a single chromosomal copy. Integration in the chromosome is catalyzed by homologous recombination using a 500-bp region identical to the *V. cholerae lacZ-lacI* genes. Integrants can be selected using the chloramphenicol resistance gene carried by the plasmid. We verified that the integration of the plasmid is lethal for *V. cholerae* cells during SOS induction using mitomycin C (MMC), and that the integration is possible in the absence of SOS induction (no MMC or using a non-cleavable LexA repressor preventing SOS induction) (Supplementary Figure S1).

To randomly interrupt *V. cholerae* genes, the pSC189 conjugative plasmid carrying a mariner transposon, its transposase and a spectinomycin resistance gene were used (44). The pTOX-SOS plasmid was introduced in the *V. cholerae* mutant library, and the clones that had recombined it into their chromosome were selected on an SOS-inducing sub-MIC of tobramycin (TOB). TOB was used at 1% of the MIC as for our previous studies (9,10). Note that while allowing us to select mutants unable to induce the SOS response, it does not allow us to select inactivated *recA* mutants, as RecA is required for plasmid integration in the chromosome through homologous recombination; in addition, we do not expect to identify hits in all SOS-regulated genes, as these genes will already be downregulated in the SOS-inactivated mutants.

**Figure 2.**
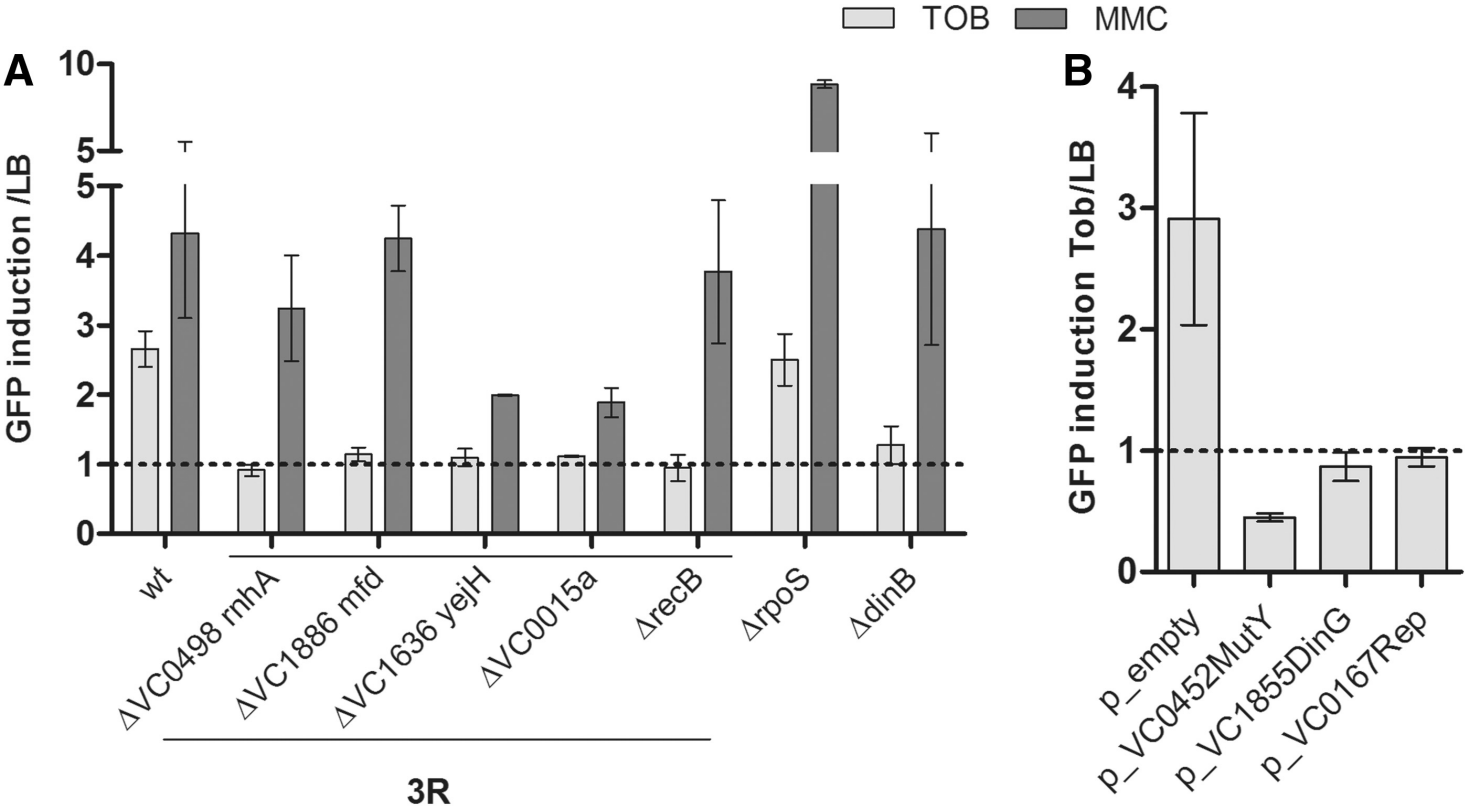
*V. cholerae* mutants unable to induce SOS after sub-MIC tobramycin treatment. GFP fused to the SOS-induced *intIA* promoter (p4640 in Table 3) was used to compare the fluorescence of various mutants with the wild-type *V. cholerae*. Histogram bars represent the ratio of GFP fluorescence in the presence of antibiotic over fluorescence in LB and thus reflect the induction of SOS by TOB or MMC. Error bars represent standard deviation. Each strain was tested at least four times. TOB was used at 0.01 µg/ml. Mitomycin C was used at 0.1 µg/ml. (**A)** Deletion of identified 3R genes (Table 1) in wild-type *V. cholerae*. WT stands for wild-type *V. cholerae.* 3R stands for replication recombination repair mutants. (**B**) Over-expression of genes identified in Table 2 in wild-type *V. cholerae*. *p_empty* stands for empty circularized pTOPO plasmid. *p_VCxxx* stands for pTOPO expressing the indicated gene.

We then identified by sequencing the location of the transposon in the mutants deficient for induction of the SOS response by sub-MIC of AGs. The interrupted genes at 1% MIC are shown in Supplementary Table S4. These mutations were found in 30 loci and belong to different functional categories, among which several were members of the 3R category (replication, recombination, repair). The other genes were classified as oxidative stress and metabolism-related, membrane proteins, motility, transcriptional regulators, guanylate cyclases and others. Thirteen of the corresponding deletion mutants chosen in each category were reconstructed in the wild-type *V. cholerae* strain and were confirmed to be deficient in SOS induction after sub-MIC TOB treatment, whereas direct DNA damage using MMC still induced the SOS response in these mutants (Supplementary Figure S2). We focused our analysis on the 3R mutants; the others will be studied and analyzed in a future report. The interrupted 3R genes at 1% MIC are shown in Table 1. Interestingly, in the search for mutants unable to induce the SOS response after sub-MIC TOB treatment, we obtained a mutant in which the *recC* gene was interrupted. The RecC protein is part of the RecBCD homologous recombination pathway that repairs dsDNA lesions. This confirms our previous observations that inactivation of *recB* results in the abolition of the sub-MIC TOB-induced SOS response (10). Additionally, the finding of this mutant constitutes a robust proof of principle of our genetic screen. The 3R deletion mutants were reconstructed in the wild-type *V. cholerae* strain. Using Green fluorescent Protein (GFP) fused to the SOS-induced *intIA* promoter (p4640 in Table 3), these mutants were confirmed to be deficient in SOS induction after sub-MIC TOB treatment, but not after treatment with the DNA-damaging agent mitomycin C (MMC) (Figure 2A). The *V. cholerae ΔrpoS* strain was used as a control known to induce SOS in response to TOB.

**Table 1.** Genes identified in the inactivation screen

Replication recombination repair		
VC2322	RecC	RecBCD double-strand break repair
^a^VC1886	Mfd helicase	Transcription repair coupling factor
^a^VC0498	RnaseH	RNA–DNA helicase
^a^VC1636	Putative (YejH_vc_)	DNA–RNA helicase
VC0015a	GreA-like	putative transcriptional regulator

^a^Gene for which deletion mutants were reconstructed and verified in wild-type *V. cholerae* N16961 *hapR+* strain.

We performed a second genetic screen using an over-expression strategy instead of an inactivation strategy: we first transformed the wild-type strain with a *V. cholerae* genomic library [expressed from the high copy number pUC19 plasmid (45)] and then selected for clones unable to induce the SOS response using the integrated pTOX-SOS system. Characterization of the surviving clones led to the identification of two *V. cholerae* genes whose over-expression prevents SOS induction by sub-MIC aminoglycoside treatment (Table 2). Recloning in pTOPO and expression from their native promoter in wild-type *V. cholerae* confirmed that the genes identified in the over-expression screen were preventing the induction of the SOS response by TOB (Figure 2B).

**Table 2.** Genes identified in the overexpression screen

Replication recombination repair			
VC1855	DinG	Helicase	
VC0452	MutY	A/G-specific adenine glycosylase-BER	

**Figure 3.**
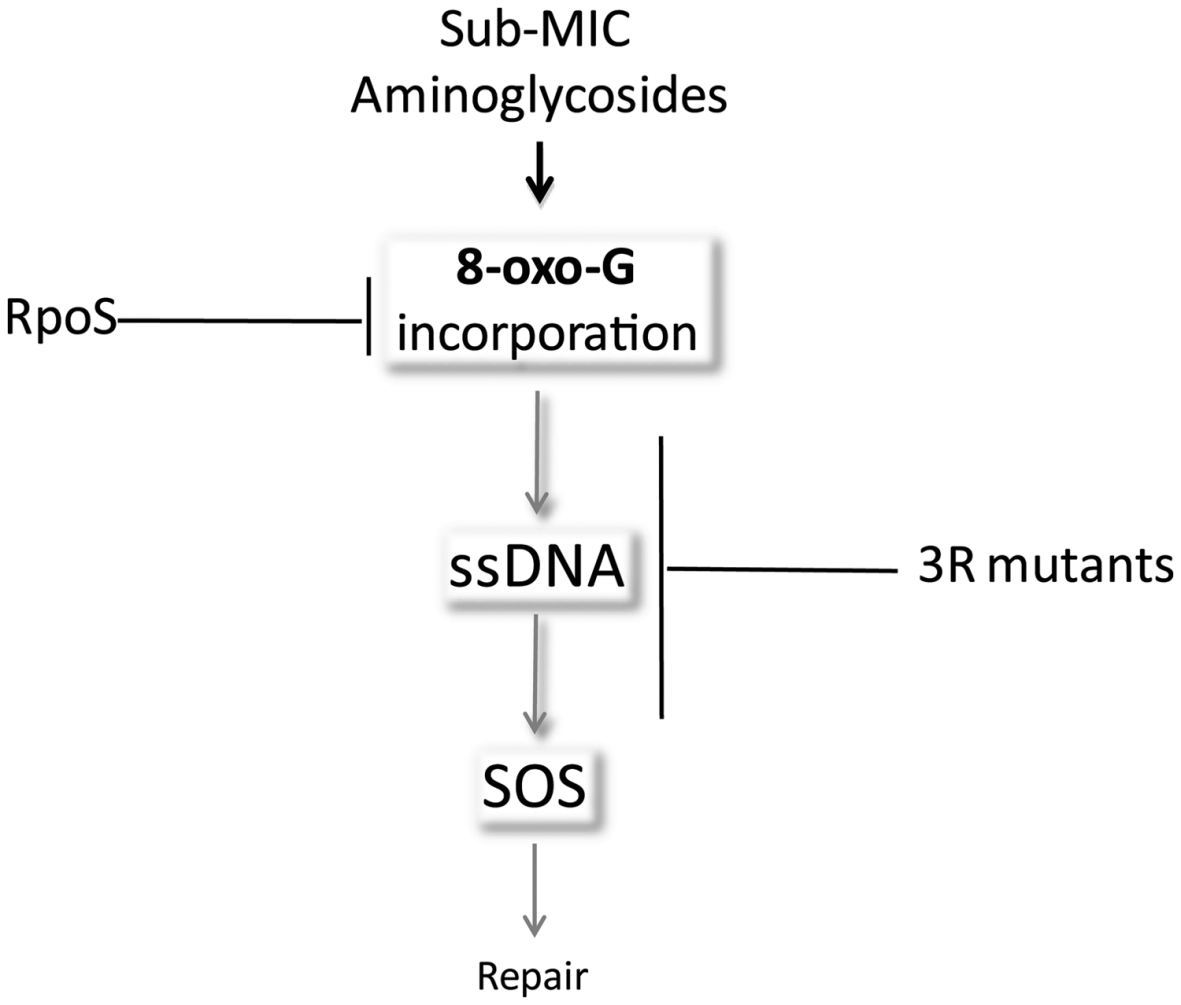
Model for SOS induction by sub-MIC aminoglycosides. Adapted from (10). In the presence of sub-MIC AGs, DNA damage is induced by 8-oxo-G incorporation or through impaired action of DNA replication and repair proteins. When present and stable, RpoS protects cells from this type of DNA damage (10). Possible implications of the 3R mutants are discussed in the text.

Our working model [as proposed in (10)] is illustrated Figure 3: in the presence of AGs, the formation of 8-oxo-G increases, leading to its incorporation in DNA. This leads to formation of ssDNA intermediates and subsequent induction of the SOS response. We hypothesized that the mutants belonging to the 3R class act after 8-oxo-G incorporation and would be involved in the formation of ssDNA. To further characterize the mechanisms of SOS induction by AGs in *V. cholerae*, we chose to focus on the possible mechanisms of such a process.

### SOS induction is triggered by oxidized DNA base incorporation and can be overcome by over-expression of the BER pathway proteins

We had previously shown that one of the mechanisms of SOS induction after sub-MIC TOB treatment involved incorporation of 8-oxo-G (10). SOS induction measurements are performed using GFP fused to the SOS-induced *intIA* promoter (p4640 in Table 3), which allows the fluorescence of various mutants to be compared with the wild-type *V. cholerae*. We further tested the effect of sub-MIC TOB in a *V. cholerae dinB* mutant. *dinB* encodes the translesional DNA polymerase PolIV, and its absence impairs the incorporation of 8-oxo-G in DNA (18). As expected, no SOS induction by sub-MIC TOB was observed in this mutant (Figure 2A). We had also shown that the over-expression of the BER pathway proteins, such as MutY, impairs SOS induction by sub-MIC AGs (10). In the search for clones unable to induce SOS after sub-MIC TOB treatment, we selected a plasmid carrying MutY (VC0452) using our over-expression screen (Figure 2B). MutY is involved in the BER of mismatches owing to 8-oxo-G incorporation in the DNA. This confirms once more our previous observations that over-expression of MutY and MutT abolishes SOS induction by TOB (10). We thus conclude that SOS induction after sub-MIC TOB treatment can be overcome by preventing 8-oxo-G incorporation in DNA and by over-expressing the BER pathway. The details of proteins involved in the induction of the SOS response by sub-MIC TOB had, however, not been further elucidated in our previous study.

**Table 3.** Strains and plasmids

Strain number	Genotype of interest	Reference or construction
*E. coli*		
MG1655	WT	Laboratory collection
A849	MG1655 *mfd::kan*	This study
7110	*dnaA46ts tna::Tn10*	Laboratory collection
B248	*rnhA::kan*	KEIO collection JW0204-2 (CSGD)
B535	MG1655 *dnaAts*	This study
B446	MG1655 *dnaAts rnhA*	This study
*V. cholerae*		
8637	N16961 *hapR+*	(9)
A072	N16961 *hapR+ recB::cm*	(10)
A321	N16961 *hapR+ rpoS::aadA1*	(10)
A126	N16961 *hapR+ mfd::aadA1*	This study (VC1886, Supplementary Table S1)
B978	N16961 *hapR+ uvrA::aadA1*	This study (Supplementary Table S1)
B742	N16961 *hapR+ mutS::aadA1*	This study (Supplementary Table S1)
A322	N16961 *hapR+ dinB::aadA1*	This study (Supplementary Table S1)
Plasmids		
p9644	pTOPO	Stratagene (circularized)
p9477	pTOPO-VC0452	This study (Supplementary Table S2)
p9437	pTOPO-VC0003	This study (Supplementary Table S2)
p9431	pTOPO-VC0004	This study (Supplementary Table S2)
p9432	pTOPO-DinG	This study (VC1855, Supplementary Table S2)
pA842	pTOPO-Pbla-Rep	This study (VC0167, Supplementary Table S2)
pB973	pTOPO-Pbla-Mfd_Ec_	This study (Supplementary Table S2)
pB258	pTOPO-Pbla-Mfd_Vc_	This study (Supplementary Table S2)
pB260	pTOPO-Pbla-VC1636	This study (Supplementary Table S2)
pC138	pTOPO-Pbla-RnhA_Ec_	This study (Supplementary Table S2)
p4640^a^	*gfp* induced by SOS, SOS reporter for flow cytometry in *V. cholerae*	(2)

^a^p4640 carrying *gfp* fused to the SOS-inducible *intIA* promoter was integrated in the chromosome of the specified *V. cholerae* strains by conjugation as described (3).

### Characterization of the 3R mutants identified in the inactivation and over-expression screens

The DinG and Rep helicases impair SOS induction by tobramycin.

The over-expression screen led to the identification of the DinG helicase. As Rep, the DinG helicase facilitates DNA replication by clearing stalled RNAPs from DNA (36). We tested the effect of Rep over-expression. We observe that, just like for DinG (VC1855), over-expression of Rep (VC0167) also impairs SOS induction by TOB (Figure 2B). This suggests that prolonged RNAP stalling can be prevented in the presence of TOB by over-expressing DinG or Rep, which would destabilize the RNAP as soon as it marks a pause.

Deletion of VC0015a, VC0498 (rnhA_vc_), VC1636 and VC1886 (mfd_vc_) relieves SOS induction by sub-MIC tobramycin in V. cholerae.

The genetic inactivation screen selecting for mutants deficient for SOS induction by AGs led to the identification of *recC_vc_*, *mfd_vc_*, *rnhA_vc_*, VC1636 and VC0015a. The effect of the RecBCD homologous recombination pathway on SOS induction by AGs in *V. cholerae* had already been characterized in our previous work (10). We deleted *mfd_vc_* (helicase), *rnhA_vc_* (RNA–DNA helicase), VC0015a (putative GreA-like protein) and VC1636 (putative RNA–DNA helicase) in *V. cholerae* and confirmed that deletion of these genes prevents the induction of the SOS response by sub-MIC TOB (Figure 2A).

The *E. coli* Mfd helicase interacts with RNAP stalled on lesions and removes it from the DNA to initiate repair (32,48). The *V. cholerae* Mfd_Vc_ shares 65% identity at the amino acid level with the *E. coli* Mfd over 75% protein of the protein length, suggesting that both proteins are orthologous and play similar roles. No other Mfd homolog could be found in the *V. cholerae* genome.

VC0498 is the ortholog of the *E coli rnhA* gene, which encodes RnaseH, and was thus named *rnhA_vc_*. This helicase degrades the R-loops formed at stalled RNAP sites in *E. coli* (24).

VC0015a is annotated as a GreA-like elongation factor. In *E. coli*, GreA stimulates the mRNA cleavage activity of the RNAP (49), and thus leads to the release of an RNAP complex that has stalled or backtracked (50) (51). VC0015a is not the *V. cholerae greA* ortholog, which is encoded by VC0634 (71% protein identity with *E. coli* GreA).

The protein encoded by VC1636 is a putative RNA–DNA helicase. Its *E. coli* ortholog, called *yejH* (60% protein identity over 97% of the protein), has never been studied. As this gene is highly conserved among bacteria, we decided to refer to VC1636 as YejH_vc_ in the following paragraphs, and to extend its functional characterization.

#### YejH_vc_ over-expression does not complement RNA degradation activity of RNaseH in RNA–DNA hybrids

**Figure 4.**
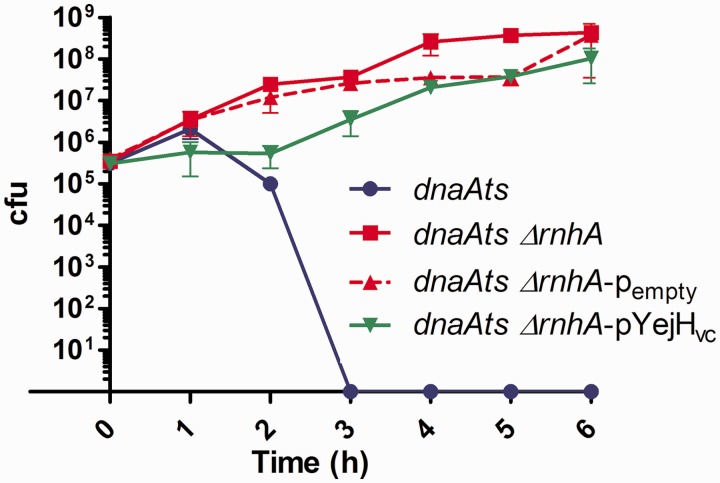
VC1636 YejH_vc_ has no RNA degrading activity at RNA–DNA hybrids when over-expressed in *E. coli*. Cells were propagated at 30°C for 1 h 30 min and were shifted to 42°C (time 0). Appropriate dilutions were plated at the indicated times and incubated at 30°C to allow growth. The Y axis shows survival at 42°C. Error bars represent standard deviation. Each strain was tested three times.

Although RNaseH and YejH do not share significant homology, the annotation of YejH_vc_ as a putative RNA–DNA helicase led us to test whether it can act like RNaseH, by degrading RNA in RNA–DNA hybrids, using the following genetic test. Replication in *E. coli* is dependent on the DnaA protein that synthesizes the RNA primers used for replication initiation by DNA polymerase III. In the absence of DnaA, no DNA replication can occur, except in *rnhA*-deleted strains, where non-degraded RNA fragments can be used as primers for the DNA polymerase. As a result, a *dnaA* thermosensitive mutant (*dnaAts*) is lethal at 42°C, as DnaA is completely inactivated, whereas a *dnaAts rnhA* mutant is viable (52,53). We hypothesized that if YejH_vc_ can degrade the RNA of RNA–DNA duplexes, its over-expression would result in the lethality of the *dnaAts rnhA* mutant at 42°C because these RNA fragments would not be able to serve as primers for DNA replication. As expected, the control strain over-expressing *E. coli rnhA* is lethal at 42°C: the plasmid expressing the wild-type *E. coli* RNaseHI was successfully cloned, but despite our efforts we were unable to transform the *dnaAts rnhA* mutant with this plasmid. Transformants systematically harbored a mutated *rnhA* in the plasmid, and we thus concluded that over-expression of RnhA in this strain prevents growth even at 30°C. On the other hand, we observed no lethal effect of the over-expression of YejH_vc_ in the *dnaAts rnhA* mutant (Figure 4, pVC1636 in green). This suggests that the YejH_vc_ protein does not degrade the RNA in RNA–DNA hybrids.

#### YejH_vc_ over-expression complements the RNAP-displacing activity of Mfd at lesions caused by UV light in E. coli

**Figure 5.**
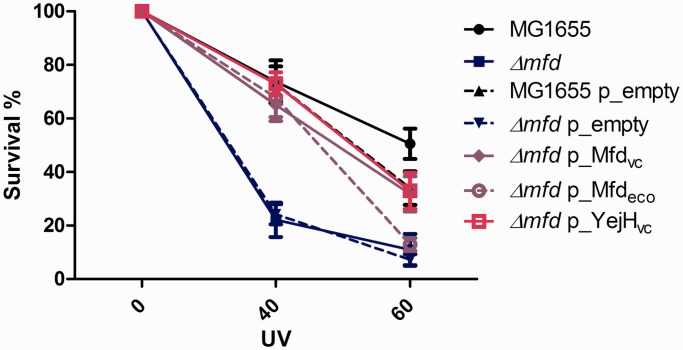
VC1636 YejH_vc_ over-expression can complement the UV sensitivity of an *E. coli Δmfd* strain. Serial dilutions of exponential cultures (OD ≈ 0.5) of different strains were plated. Plates were UV irradiated at 0/40/60 J/m^2^ and incubated for 24 h at 37°C. The ratios of the numbers of colonies on irradiated plates to those on non-irradiated plates were calculated. The Y axis shows survival, i.e. the ratio of colony-forming units (*cfu*) at the indicated UV dose over *cfu* of non-irradiated culture. Error bars represent standard deviation. Each strain was tested at least three times.

As mentioned earlier, Mfd recognizes and binds the RNAP stalled at a site of DNA damage and releases the RNA transcript and the RNAP enzyme from DNA, to recruit the NER machinery (30–32). It is known that the deletion of *mfd* renders *E. coli* cells sensitive to UV irradiation (54). Our results show that the *V. cholerae mfd* (VC1886) gene can effectively complement the UV sensitivity caused by the *Δmfd* mutation in *E. coli* at 40 J.m^−^^2^ and 60 J.m^−^^2^ (Figure 5). As YejH_vc_ is a putative helicase, we addressed whether it could be involved in interactions with RNAP at the site of lesions. We tested whether YejH_vc_ (VC1636) could complement UV damage repair in *Δmfd* cells, postulating that if YejH_vc_ can act like Mfd by destabilizing the RNAP stalled on DNA lesions, destabilization of the RNAP by YejH_vc_ in a *Δmfd* mutant would lead to the recovery of UV resistance. We found that YejH_vc_ (pVC1636) restores UV resistance to a *Δmfd E. coli* mutant (Figure 5), whereas YejH_vc_ over-expression does not change the UV resistance profile of the wild-type *E. coli* strain (Supplementary Figure S3). We thus conclude that YejH_vc_ has a function similar to that of Mfd at UV lesions in *E. coli,* even though it is not an ortholog of *mfd.*

#### The absence of mfd_vc_, rnhA_vc_, rep and yejH_vc_ causes loss of viability in the presence of sub-MIC tobramycin in V. cholerae

The identification of genes involved in mechanisms related to RNAP destabilization (*mfd*, *rnhA*, *rep* and *dinG*) is interesting. Together with the putative *greA*-like gene, they constitute a clue suggesting that RNAP could be stalled on DNA lesions formed by a treatment with sub-MIC TOB, and that the mechanisms of RNAP restart or removal involve the formation of ssDNA, which induces the SOS response.

**Figure 6.**
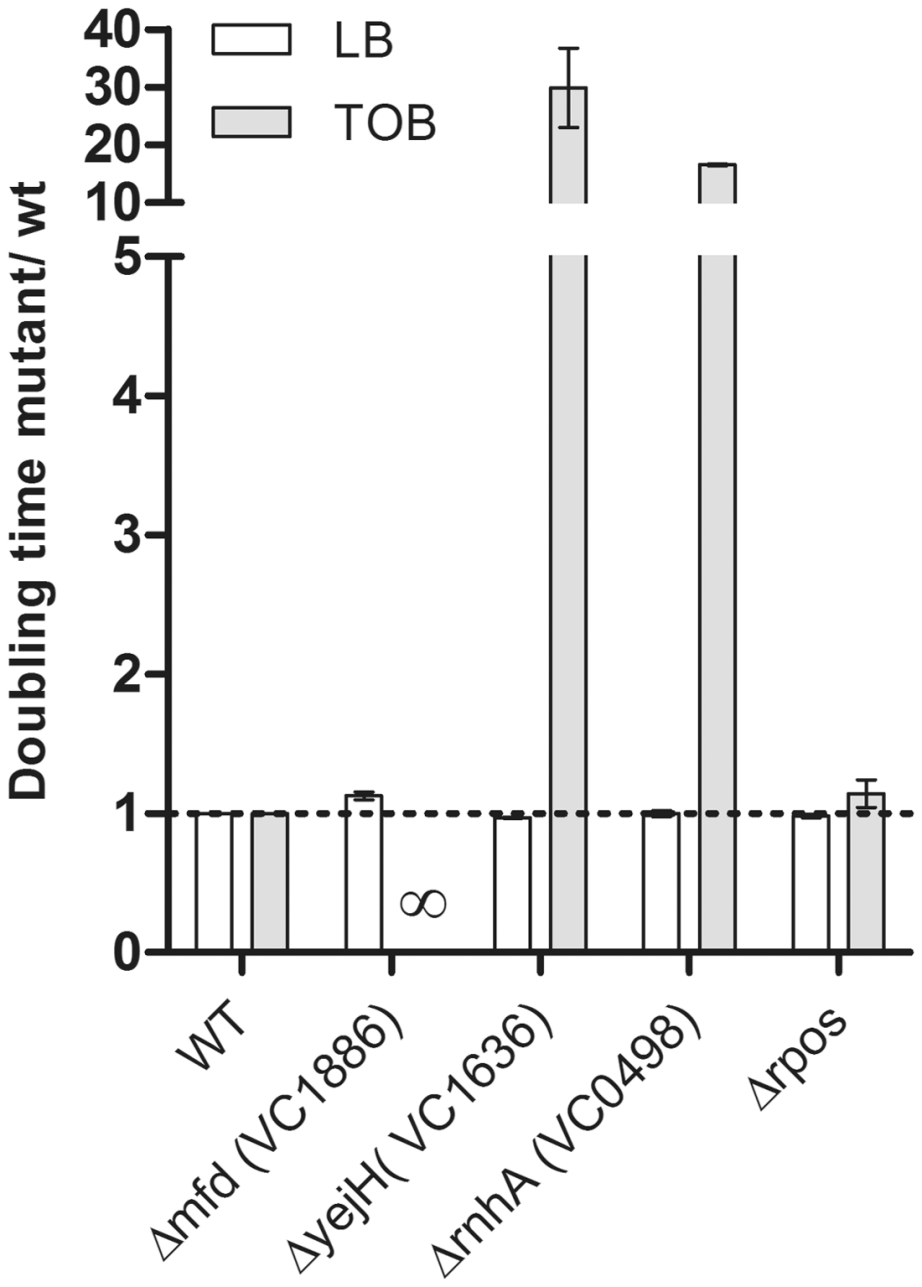
Impact of 3R gene inactivations in *V. cholerae* on growth in sub-MIC tobramycin. Histogram bars represent the ratio of doubling time of the mutant over isogenic wild-type strain in LB or TOB, used at 0.25 µg/ml, which corresponds to 25% of the MIC. *ΔrpoS* where SOS is induced by TOB was used as control. ∞ means that bacteria did not grow. Error bars represent standard deviation. Each strain was tested at least three times.

We hypothesized that the RNAP stalled on a DNA lesion could prevent the lesion from being repaired. Repair is often associated with transient formation of ssDNA (and RecA polymerization). Thus, the RNAP stalled on a lesion could prevent induction of the SOS response by preventing this transient formation of ssDNA. We tested the effect of a TOB treatment on the viability and growth rates of mutants of *mfd_vc_*, *rnhA_vc_* and *yejH_vc_* and compared them with the wild-type strain (Figure 6). Because the mutants were isolated at 1% of MIC (TOB 0.01 µg/ml), we tested their viability at a higher concentration, i.e. 50% of MIC (TOB 0.5 µg/ml), a concentration at which the wild-type *V. cholerae* strain shows no growth defect. All strains grew well in LB without antibiotic; however, we found that the 3R mutants have a strong growth defect in the presence of TOB at 50% MIC (Figure 6). The *mfd* mutant did not grow in a liquid culture at TOB 0.5 µg/ml, and the growth of *yejH* and *rnhA* mutants was 10–20 times slower. This was consistent with our hypothesis postulating that inactivation of *rnhA* or *mfd* leads to prolonged RNAP stalling and reduced growth rate (due to reduced repair). Moreover, we determined the MIC of tobramycin on agar plates in these mutants: while the MIC is of 1 µg/ml for the wild-type *V. cholerae*, it is reduced to 0.70 µg/ml for the *mfd* mutant and 0.85 for the *rnhA* and *yejH* mutants, meaning that these mutants are more sensitive to tobramycin than the wild-type strain. Our results suggest that RnaseH_vc_, Mfd_vc_ and YejH_vc_ help dislodge RNAP stalled at 8-oxo-G lesions formed after aminoglycoside treatment in *V. cholerae*, creating ssDNA intermediates that can be bound by RecA. Conversely, the action of DinG and Rep does not seem to involve ssDNA, as their over-expression prevents induction of the SOS response (Figure 2B), suggesting that they rather prevent the RNAP from stalling.

### Inactivation of NER does not relieve SOS induction by sub-MIC tobramycin in *V. cholerae*

**Figure 7.**
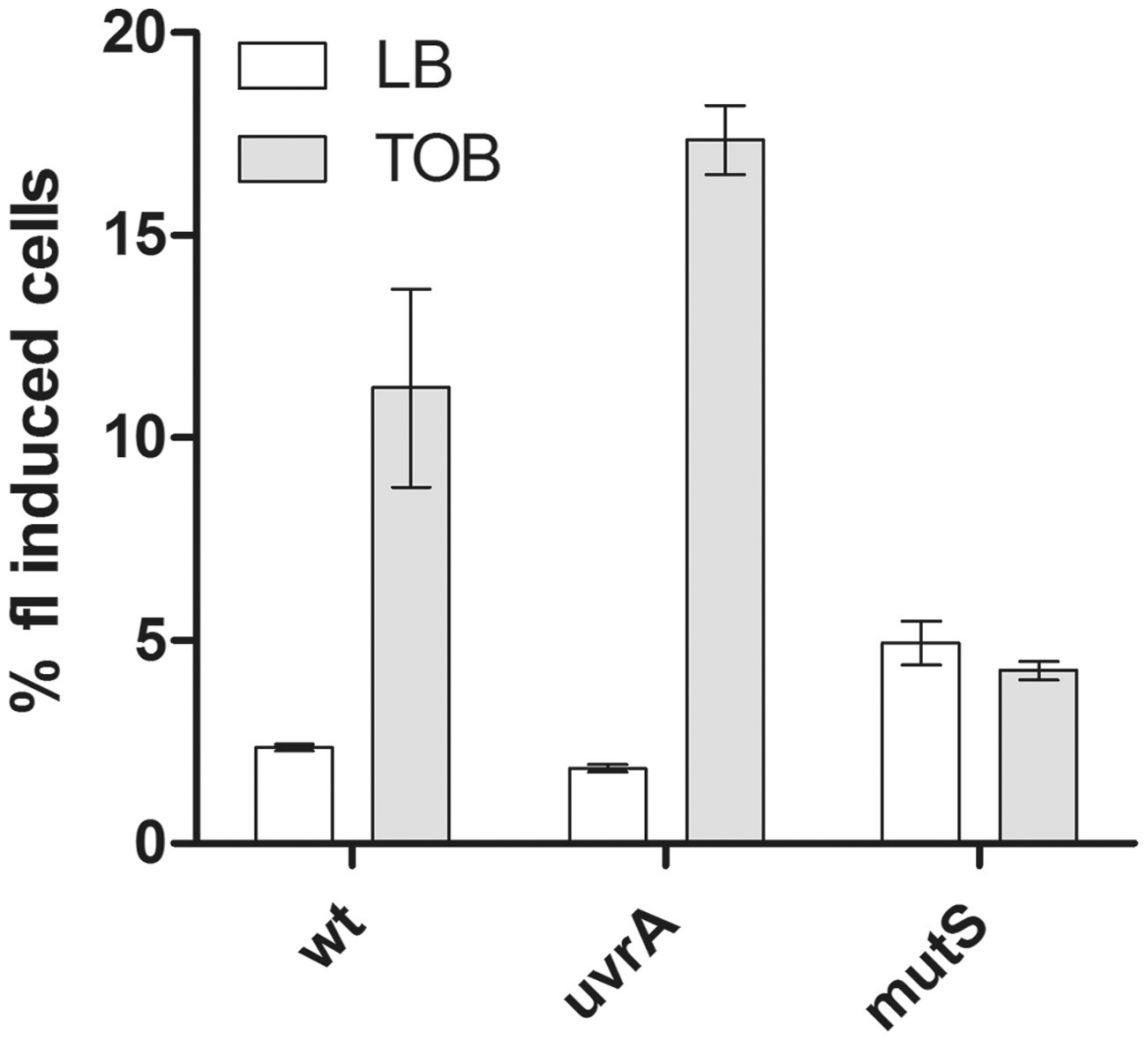
Role of the NER and MMR pathways on sub-MIC tobramycin mediates SOS induction. Histogram bars represent the percentage of GFP fluorescence (i.e. SOS induction) in indicated *V. cholerae* strains in LB or sub-MIC TOB. *gfp* fusion with the SOS-induced promoter of *intIA* is carried by plasmid p4640 as described in Table 3. TOB was used at 0.01 µg/ml. Error bars represent standard deviation. Each strain was tested at least three times.

As explained above, our hypothesis was that the RNAP stalled on a lesion could prevent SOS induction by preventing transient ssDNA formation. This brought us to the next question: which repair mechanisms lead to ssDNA formation during repair of lesions caused by sub-MIC TOB? We know that over-expression of the BER proteins leads to a decrease in the induction of the SOS response in these conditions [the present study and (10)], suggesting that ssDNA does not originate from the BER pathway. We tested whether ssDNA intermediates that induce SOS in the presence of TOB are formed during the NER pathway. If SOS induction by sub-MIC TOB is relieved in the *V. cholerae mfd* mutant due to uncoupling of transcription stalling and NER, then inactivation of NER should also prevent SOS induction. We constructed a *V. cholerae uvrA* mutant. In this mutant, where one can assume that NER is abolished, we observed that SOS is still induced by sub-MIC TOB (Figure 7). We conclude that SOS induction by TOB involves another repair pathway.

### Inactivation of *mutS* abolishes induction of the SOS response by a sub-MIC of tobramycin in *V. cholerae*

Because the 8-oxo-G incorporated in DNA can pair with adenines instead of cytosines, thus forming a mispair, we tested the effect of the inactivation of the MMR pathway on induction of the SOS response. We constructed a *V. cholerae mutS* mutant, in which MMR is abolished, and we observed that SOS induction by sub-MIC of TOB is also abolished (Figure 7). The reason why we did not obtain this mutant in our genetic screen could be because this mutant grows slowly in the presence of TOB. The observation that the SOS response is abolished in *V. cholerae mutS* suggests its induction by TOB can occur through the formation of dsDNA breaks and break repair by the MMR pathway (55). Alternatively, the action of MutS could be independent of the MMR pathway, as *E. coli* Mfd-mediated TCR (but not NER) was abolished in a *mutS* mutant (56).

Collectively, these results suggest that destabilization of the RNAP stalled at 8-oxo-G lesions are repaired through the formation of ssDNA intermediates that induce the SOS response. The formation of ssDNA under these conditions requires the presence of MutS. Preventing the induction of SOS and, consequently, of DNA repair negatively affects growth in the presence of TOB.

## DISCUSSION

We have previously shown that the incorporation of oxidized guanine in DNA is involved in the induction of the SOS response by sub-MICs of the aminoglycoside tobramycin and that the RpoS-dependent stress response prevents this SOS response (10). To have further insight into mechanisms connecting the incorporation of 8-oxo-G and induction of the SOS response by AGs, we developed a genetic screen and identified *V. cholerae* mutants that do not induce the SOS response on exposure to sub-MICs of AGs but that are still proficient for its induction in other conditions.

Our genetics screen led to the identification of several ‘3R mutants’. First, we identified RecBCD, involved in homologous recombination and in the repair of double-strand breaks, that had already been identified as necessary for the induction of the SOS response by sub-MIC AGs (10). Interestingly, AGs affect translation, and mistranslational corruption of DNA polymerase III was observed to lead to replication fork collapse, which requires RecABCD and the SOS response for repair (57). Moreover, translation inactivation using an aminoglycoside was shown to increase the formation of R-loops, which is usually inhibited by ribosomes (23). Double-strand breaks were also shown to result from R-loops in some cases (23).

In light of the results presented above, we propose that the RNAP can pause transiently when it encounters 8-oxo-G. Subsequently, it is either dislodged without formation of ssDNA intermediates or stalls for a prolonged period.

**Figure 8.**
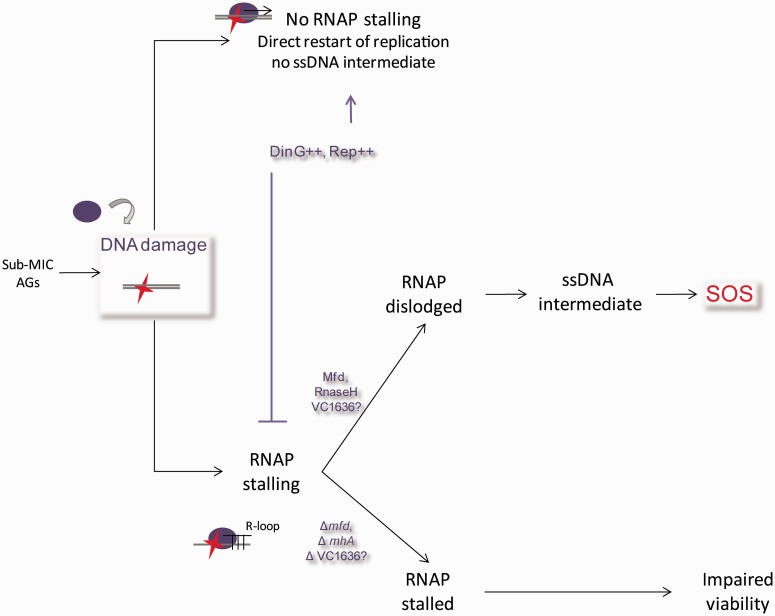
Model proposed for the involvement of RNAP in SOS induction after sub-MIC AG treatment. Discussed in the text.

In the first scenario, the RNAP that has paused can be destabilized by the DinG or Rep helicases (36), when they are over-expressed (Figure 8), and the replication then proceeds without being blocked but can be mutagenic (GC to TA transversions). Direct restart of the replication fork can take place after displacement of the stalled RNAP, as the replisome is stable enough to wait for the stalled RNAP to be dislodged (58). In the case where RNAP does not stall, the BER machinery can be recruited at the site of 8-oxo-G lesions. The SOS response would not be induced in this case, as no subsequent repair is necessary (59).

In the second case, we propose that prolonged RNAP stalling prevents the formation of ssDNA intermediates necessary for the induction of SOS. Prolonged RNAP stalling leads to the formation of a stable DNA–RNA hybrid (R-loop) at the site of the lesion (60). R-loop formation causes replication fork arrest and genomic rearrangements (22). Moreover, R-loops can prime origin-independent replication fork initiation. RNaseH is able to recognize and unwind such R-loops, and we propose that this leads to the formation of an ssDNA region prone to induce the SOS response (Figure 8). However, it should be noted that two different *rnhA* genes are present in the *V. cholerae* genome. The VC0498 RNaseH that was identified in our screen has only 40% identity with the *E. coli* RNaseH protein. We cannot exclude that the VC0498 RNaseH can have an additional or a different function than the *E. coli* RNaseH or the other *V. cholerae* RNaseH VC2234 (75% identity with *E. coli* RNaseH).

On the other hand, the Mfd helicase can bind DNA–DNA or DNA–RNA duplexes [reviewed in (11)]. Mfd recognizes the RNAP stalled at a site of DNA damage and releases the RNAP enzyme from the DNA, which allows recruitment of the NER machinery (30,31,61), leading to NER. Mfd thus couples NER to the stalled RNAP. However, we have shown here that Mfd-dependent SOS induction does not require the presence of UvrA, which excludes transcription-coupled NER as the origin of SOS-inducing ssDNA intermediates.

In the presence of Mfd, we propose that the arrested RNAP is removed and the R-loop remains, as it was observed in non-replicating *E. coli* cells where R-loops accumulate upon Mfd action (23). Such R-loops can prime origin-independent replication initiations. It was proposed that ssDNA stretches, which are SOS inducers, are formed from dsDNA ends through RecBCD action when the R-loop primed replication fork hits a nick and collapses. This is also consistent with our observations that RecBCD is involved in the induction of SOS by AGs. Moreover, such nicks (or single-stranded gaps) can be formed upon repair of 8-oxo-G incorporated in DNA. When stalled RNAP is removed by Mfd, the remaining R-loop may thus initiate a replication fork that would collapse when encountering the gap forming a dsDNA break. This would induce SOS through the RecBCD recombination pathway. The model proposed by Wimberly *et al.* links Mfd and RecBCD to the formation of ssDNA without the need for UvrA, and hence seems consistent with our results.

In the absence of Mfd, it has been observed that the template strand can be protected from repair by stalling the RNAP (62), highlighting the importance of Mfd for the repair of certain lesions. Mfd-dependent TCR preferentially targets the repair of bulky DNA damage (27). In addition, 8-oxo-G is not a bulky lesion and does not necessarily block transcription or DNA replication, and 8-oxo-G lesions can actually be efficiently bypassed by RNAP after a transient pause (63,64). Thus, it is possible that in the absence of Mfd, the RNAP bypasses this type of lesion without inducing the SOS response. Alternatively, a blocked RNAP can be eventually removed by diffusion in the absence of Mfd, but this requires too much time in conditions of stress and significantly affects cell viability. This is what we observe in the presence of sub-MIC TOB.

Interestingly, Mfd has also been reported to generally play a role in recombination in *Bacillus subtilis* (65) likely because it prepares the DNA substrate, such as ssDNA for RecA polymerization for recombination enzymes, highlighting a possible role in induction of the SOS response. Mfd is also required for the repair of dsDNA breaks (caused by MMC) in *Helicobacter pylori,* where an *mfd* mutant is susceptible to antibiotics and has a DNA repair defect (66). Mfd is involved in increased mutation frequencies in *Campylobacter jejuni* (67). Moreover, Mfd promotes genetic diversity in non-growing stressful conditions in *Bacillus subtilis* (68). These observations of an Mfd-dependent increase of resistance development and genome plasticity can in fact be explained by the induction of the SOS response in the presence of Mfd, as described in the present study. Interestingly, *mfd* inactivation was also identified in the similar screen using TOB 10% MIC as well as at 1% MIC of other antibiotics such as tetracycline and the AG neomycin (data not shown).

On the other hand, we identified a role for MutS in the induction of SOS by tobramycin. MutS belongs to the MMR pathway. Unlike for NER, MMR does not repair lesions but rather mismatches that are formed after replication: the MutL and MutS proteins recognize the mismatch, MutH nicks the unmethylated (i.e. new) DNA that is then removed by UvrD and the gap is filled by resynthesis. Interestingly, RpoS expression is known to decrease MutS levels (69). Strikingly, Mfd-mediated TCR (but not NER) was observed to be abolished in an *E. coli mutS* mutant (56), which can explain our observation that SOS induction by TOB is abolished in a *mutS* mutant. We know that low RpoS levels lead to SOS induction in the presence of AGs (10). We can speculate that the RpoS-dependent protection from SOS induction described previously (10) is not only a protection from oxidative stress, as we have already proposed, but also a reduced Mfd action in *E. coli*, due to reduced levels of MutS. When RpoS levels are decreased, MutS levels are increased, which could increase Mfd-dependent SOS induction.

Finally, we identified YejH_vc_, a previously uncharacterized RNA–DNA helicase, highly conserved among gamma-proteobacteria, and possibly involved in the removal of blocked RNAP during replication fork progression. We show for the first time that YejH_vc_ is functional in *V. cholerae*, as its deletion prevents SOS induction by AGs and its over-expression restores UV resistance in an *mfd* mutant in *E. coli*. YejH_vc_ is conserved among prokaryotes, and protein blast shows that YejH_vc_ presents homologies with the human ERCC3 protein (Rad25 in yeast). Rad25 and ERCC3 are components of the eukaryotic transcription factor TFIIH involved in transcription initiation and NER (70,71). ERRCC3 was found to be involved in genetic diseases and cancer (72,73). Further work is underway to biochemically and genetically characterize the actions of purified YejH_vc_ and YejH_Ec_ proteins and their possible action in transcription-coupled NER and interactions with RNAP. VC0015a greA-like protein (which does not have a homolog in *E. coli*) may have an RNAP destabilization activity under certain conditions that also has to be characterized.

Aminoglycosides target protein translation, but they clearly also have a negative effect on transcription and replication at sub-MIC. The transcription elongation rate is controlled by translating ribosomes: active ribosomes can prevent RNAP backtracking (40,74). AGs may interfere with this mechanism and enhance the rate of RNAP backtracking and thus RNAP–DNAP collisions and replication fork collapse. The origin of 8-oxo-G incorporated in DNA remains to be discovered. One explanation could be production of reactive oxygen species (ROS) in *V. cholerae* in the presence of low concentrations of aminoglycosides. Alternatively, mistranslation and subsequent misfolding of proteins due to the aminoglycoside action could lead to increased oxidation of proteins by a regular amount of intracellular ROS created during respiration as shown previously (75). Such protein oxidation can be the origin of an oxidative stress leading to guanine oxidation (76).

The link between bactericidal antibiotics, ROS formation and cell death has lately been subject to debate. To relieve possible confusion, it may be worth mentioning that the work described here was performed using low concentrations of aminoglycosides, which do not hamper bacterial growth. On the other hand, striking studies presented ROS formation as the key step leading to cell death by beta-lactams, fluoroquinolones and aminoglycosides (77,78). This study by Kohanski *et al.* suggests that all bactericidal antibiotics, regardless of their cellular target, have the potential to induce ROS formation, which kills bacteria. Subsequent studies challenged this hypothesis, as no cell death due to ROS formation occurred using these antibiotics in different experimental conditions (79,80). Finally, a recent report elegantly demonstrates that in *E. coli* it is not ROS that kill bacteria upon AG treatment but rather increased AG uptake due to differential intracellular iron levels and synthesis of iron–sulfur clusters (81). In these latter studies (77,79–81), antibiotics were used at lethal concentrations because the lethality of antibiotics was what the authors wanted to address. Conversely, in our experiments, we test SOS induction, regardless of cell death, using concentrations 100-fold below the MIC. Hence, this study does not address lethality but only SOS induction by antibiotics.

Overall, our previous and present data highlight the fact sub-MIC antibiotics may be potent stressors for bacteria and that SOS DNA damage response and RpoS general stress response synergistically protect cells from this kind of insults.

## SUPPLEMENTARY DATA

Supplementary Data are available at NAR Online, including [82–99].

## FUNDING

Institut Pasteur, the Centre National de la Recherche Scientifique [CNRS-UMR3525]; the European Union Seventh Framework Programme [FP7-HEALTH-2011-single-stage] ‘Evolution and Transfer of Antibiotic Resistance’ (EvoTAR); and the French Government’s Investissement d'Avenir program Laboratoire d'Excellence ‘Integrative Biology of Emerging Infectious Diseases’ [ANR-10-LABX-62-IBEID]. Z.B. is supported by a DIM Malinf postdoctoral fellowship (Conseil régional d'Île-de-France) and EvoTAR. Funding for open access charge: Institut Pasteur.

*Conflict of interest statement*. None declared.

## Supplementary Material

Supplementary Data
