# Supplementary material for: Identification of genes involved in low aminoglycoside-induced SOS response in Vibrio cholerae: a role for transcription stalling and Mfd helicase
Source: Nucleic Acids Res. 2013 Dec 5;42(4):2366–79. doi: 10.1093/nar/gkt1259 (PMC3936754; doi:10.1093/nar/gkt1259)
Supplement: Supplementary Data [file supp_42_4_2366__index.html]

Identification of genes involved in low aminoglycoside-induced SOS response in Vibrio cholerae: a role for transcription stalling and Mfd helicase — Identification of genes involved in low aminoglycoside-induced SOS response in Vibrio cholerae: a role for transcription stalling and Mfd helicase — Supplementary Data 

# Identification of genes involved in low aminoglycoside-induced SOS response in *Vibrio cholerae*: a role for transcription stalling and Mfd helicase

## Supplementary Data

files

**Files in this Data Supplement:**

- Supplementary Data - pdf file
