# Supplementary material for: Identification of genes involved in low aminoglycoside-induced SOS response in Vibrio cholerae: a role for transcription stalling and Mfd helicase
Source: Nucleic Acids Res. 2013 Dec 5;42(4):2366–79. doi: 10.1093/nar/gkt1259 (PMC3936754; doi:10.1093/nar/gkt1259)
Supplement: Supplementary Data [file supp_gkt1259_nar-02817-d-2013-File010.pdf]

Supplementary Data

Supplementary figure legends

Figure S1. pTOX plasmid is toxic when SOS is induced. pTOX was conjugated into wild type *V. cholerae* N16961hapR+ or isogenic *lexAind* strains. *lexAind* is a non cleavable allele of the SOS repressor LexA and this strain is thus constitutively repressed for SOS. **A:** Histogram bars represent integration frequency measured by counting chloramphenicol resistant colonies which have pTOX integrated in their chromosome over total *cfu*. In the absence of SOS induction (no MMC) pTOX integration frequency is about 2.10^{-5} in the wild type strain. Upon SOS induction (MMC), integration frequency drops to 10^{-7}. In *lexAind* mutant, pTOX integration frequency is about 5.10^{-6} (which is expected due to lower general homologous recombination efficiency in *lexAind* strains). Addition of MMC in this strain does not affect pTOX integration, showing that when SOS cannot be induced, pTOX integration is possible, whereas upon SOS induction, pTOX integration is not observed (~10^{-7}). **B**: same experiment represented as % of integration in MMC compared to LB.

Figure S2. *V. cholerae* mutants unable to induce SOS after sub-MIC tobramycin treatment. Histogram bars represent the ratio of GFP fluorescence in the presence of antibiotic over fluorescence in LB and thus reflect the induction of SOS by TOB or MMC. Error bars represent standard deviation. Each strain was tested at least 4 times. TOB was used at 0.01µg/ml. Mitomycin C was used at 0.1µg/ml. Deletion of identified genes (Table S4) in wild type *V. cholerae*. WT stands for wild type *V. cholerae.* 3R mutants are shown in Figure 2 only. *Δrpos* was used as control known to induce SOS in response to TOB. *ΔrecB* was used as control known not to induce SOS in response to TOB

Figure S3. VC1636 $YejH_{vc}$ **over-expression does not have any effect on the UV sensitivity of wild type *E. coli.*** Serial dilutions of exponential cultures (OD ≈ 0.5) of different strains were plated. Plates were UV irradiated at 0/40/60 J/m^2 and incubated for 24 h at 37°C. The ratios of the numbers of colonies on irradiated plates to those on non irradiated plates were calculated. The Y axis shows survival, i.e. the ration of colony forming units (*cfu*) at the indicated UV dose over *cfu* of non irradiated culture. Error bars represent standard deviation. Each strain was tested 3 times.

Supplementary Tables

Table S1: 3-step PCR assembly for gene inactivation in *V. cholerae*

Insertion of *aadA1* for inactivation of	Forward region on *V. cholerae* chromosome	*aadA1* amplification on pAM34	Reverse region on *V. cholerae* chromosome	Strain number pTOPO	Strain number *V. cholerae* Δ
VC0498 *rnh*	**ZB21**/ZB22	ZB25/ZB26	ZB23/**ZB24**	A866	A898
VC1886 *mfd*	**2025**/2026	2029/2030	2027/**2028**	A125	A126
VC1636 *yejH*	**ZB15**/ZB16	ZB19/ZB20	ZB17/**ZB18**	A865	A897
VC 0535 *mutS*	**2537**/2538	2541/2542	2539/**2540**	B740	B742
VC0394 *uvrA*	**2794/**2795	2798/2799	2796/**2797**	B976	B978
VC2287 *dinB*	**2212/**2213	2216/2217	2214/**2215**	A324	A322

The 3 PCR products were assembled as "Forward region-*aadA1*-Reverse region" using oligonucleotides indicated in bold.

Table S2 : Plasmid constructions for over-expressions

Amplified gene	Oligonucleotides	Strain number pTOPO
own promoter-VC0452	1586/1587	p9477
own promoter-VC1855	1575/1576	p9432
P*bla*-VC0167	ZB3/ZB4	pA842
Pbla-Mfd_{Ec}	2664/2665	pB973
Pbla-Mfd_{Vc}	Z35/Z36	pB258
Pbla-VC1636	Z39/Z40	pB260
Pbla- $RnhA_{Ec}$	2870/2871	pC138

Table S3: Oligonucleotides used in this study.

Name	Sequence 5' - 3'
**ZB3**	TGTAAGTTTATACATAGGCGAGTACTCTGTTATGGATGCGTCTAAACCCCGGCCAACAACAAGC
**ZB4**	TTATTTCCCTCGTTTTGCCGCCATCATCGC
**ZB15**	CAAACATACGCTTCACTTGGTGATACTTGCCTTCATGG
**ZB16**	GCGAGCATCGTTTGTTCGCCCAGCTTCTGTATGGAACGGGTCAACTTCTCAAAAGTGGTGAAGTGACCGTCGATGATGTGG
**ZB17**	CGTGAAAGGCGAGATCACCAAGGTAGTCGGCAAATAATGTCAGCTGCAGCGGTATTAGCTACGTTCGCTCACCCC
**ZB18**	GCCCATTCAACTGCTGATCGACCGTGGCATAGAGTTCTGC
**ZB19**	CACATCATCGACGGTCACTTCACCACTTTTGAGAAGTTGACCCGTTCCATACAGAAGCTGGGCGAACAAACGATGCTCGC
**ZB20**	GTGATTGGGGTGAGCGAACGTAGCTAATACCGCTGCAGCTGACATTATTTGCCGACTACCTTGGTGATCTCGCCTTTCACG
**ZB21**	TCAATCATTTTGTTTAACAACCGAGGGAACGATGG
**ZB22**	GCGAGCATCGTTTGTTCGCCCAGCTTCTGTATGGAACGGGTTTTTTATCTCTCTAATTGTAAAAAGGGCGCATGACTTTGC
**ZB23**	CGTGAAAGGCGAGATCACCAAGGTAGTCGGCAAATAATGTCTACAAGAATTCATAGTTTGGGAGGGTAACACCTCTCAAGC
**ZB24**	GAGAAAGCCAATTGGTTGGGCGATGTCAGTCTCGCCCGATAGC
**ZB25**	CAAAGTCATGCGCCCTTTTTACAATTAGAGAGATAAAAAACCCGTTCCATACAGAAGCTGGGCGAACAAACGATGCTCGC
**ZB26**	GCTTGAGAGGTGTTACCCTCCCAAACTATGAATTCTTGTAGACATTATTTGCCGACTACCTTGGTGATCTCGCCTTTCACG
**Z35**	TGTAAGTTTATACATAGGCGAGTACTCTGTTATGGATGACTACACACTCTTTACTCTCTCTGTTTTCG
**Z36**	TTACCGAGCAGGTAGTACATTTTGCTGGAAATTCTTCAACATGTCAGCG
**Z39**	TGTAAGTTTATACATAGGCGAGTACTCTGTTATGGGTGGCCTCTTTGCGCGTTGTACCTAGCG
**Z40**	TCAAGTAGGAATGGTATAAGCTCGGTTTGATAAGTGAGCGTCGG
**1273**	ATTCGGGATCCCTTTACTGTATAAAGAAACAGTATAAACTGTTTAAACATACAGTATTGGTTAATCATACAGGTGCAAACATGCATGTGAAACCAGTAACGTTATACGATGTCG
**1274**	GTCAACTAGTCTCACTGCCCGCTTTCCAGTCGGGAAACC
**1275**	TTTACACTTTATGCTTCCGGCTCGTATGTTGTGTGGAATTGTGAGCGGATAACAATTTCACACAGGAAACAGCTATGCGCATGAGTGGTGACGTTATGAGAAATCAATATAATACACAAG
**1276**	TTAAAATACTCGGTATGAATCAGAAAAAAGACCGTATTTATCGG
**1369**	ATTCGGGATCCCTTTACTGTATAAAGAAACAGTATAAACTGTTTAAACATACAGTATTGGTTAATCATACAGGTGCAAACATGCATGTGAAACCAGTAACGTTATACGATGTCG
**1370**	GTCAACTAGTCTCACTGCCCGCTTTCCAGTCGGGAAACC
**1575**	GCGCAATCCTAATTGTGGTGAGTTAATCG
**1576**	CACAGGATTACAACCCGACAGAAAACCAGAATAAAACTCC
**1586**	CTATTGCCTGCACATTACTAAATCAGAGC
**1587**	GATTGCCGTTATTTTCAGCAAACTGCGGG
**2025**	CACCACGCGCTAACCATCAATGTGG
**2026**	GCGAGCATCGTTTGTTCGCCCAGCTTCTGTATGGAACGGGAGTCACGCTTGAGGTTTTTCTCGGTCTAGTGAGCGTTGACGC
**2027**	CGTGAAAGGCGAGATCACCAAGGTAGTCGGCAAATAATGTCGATTCTCAAATTGATAGGACAGGTAAGCGGCAAATCAGTACG
**2028**	CGGATTGGCGACCCTGATCATTTTGACGCC
**2029**	GCGTCAACGCTCACTAGACCGAGAAAAACCTCAAGCGTGACTCCCGTTCCATACAGAAGCTGGGCGAACAAACGATGCTCGC
**2030**	GCGTACTGATTTGCCGCTTACCTGTCCTATCAATTTGAGAATCGACATTATTTGCCGACTACCTTGGTGATCTCGCCTTTCACG
**2212**	CTGGTCAACTGCTCACGATACTGACGTACC
**2213**	GCGAGCATCGTTTGTTCGCCCAGCTTCTGTATGGAACGGGGCCGATGCCTCTCTTAAAACAGACATCATGGAGTGGGG

**2214**	CGTGAAAGGCGAGATCACCAAGGTAGTCGGCAAATAATGTCAGCCCACCAATACTGTATACATAAACAGTATAATAATAAGC
**2215**	GACAGGCTTGATGGTCATGGGCGAAGAGC
**2216**	TATCACCCCACTCCATGATGTCTGTTTTAAGAGAGGCATCGGCCCCGTTCCATACAGAAGCTGGGCGAACAAACGATGCTCGC
**2217**	CTTATTATTATACTGTTTATGTATACAGTATTGGTGGGCTGACATTATTTGCCGACTACCTTGGTGATCTCGCCTTTCACG
**2537**	CGGCATTTTTGATGCTGCACGAGTCGATCAGG
**2538**	GCGAGCATCGTTTGTTCGCCCAGCTTCTGTATGGAACGGGAATCTTATGTCGCTGCTTATCATCATCTGTGC
**2539**	CGTGAAAGGCGAGATCACCAAGGTAGTCGGCAAATAATGTCTTATTGCCCATATCTCAAGCATGGAATCTACAGATTCC
**2540**	TAGACCGACCTGTCGATGATGTCACTAAGATGCTGCG
**2541**	GCACAGATGATGATAAGCAGCGACATAAGATTCCCGTTCCATACAGAAGCTGGGCGAACAAACGATGCTCGC
**2542**	GGAATCTGTAGATTCCATGCTTGAGATATGGGCAATAAGACATTATTTGCCGACTACCTTGGTGATCTCGCCTTTCACG
**2664**	TGTAAGTTTATACATAGGCGAGTACTCTGTTATGGATGCCTGAACAATATCGTTATACGCTGC
**2665**	TTAAGCGATCGCGTTCTCTTCCAGTTCACGC
**2794**	CACGGCTTCAATGACGCTACCCAGTAGAATAAACC
**2795**	GCGAGCATCGTTTGTTCGCCCAGCTTCTGTATGGAACGGGAGAAAAAAAGGCTAAAGTAATGAAACCAGCG
**2796**	CGTGAAAGGCGAGATCACCAAGGTAGTCGGCAAATAATGTCCTGGGTTGCTCTTTGCTAAACGAAGTGC
**2797**	TGATCGCAGGTGAGATGATCAAAGACG
**2798**	TTCATTACTTTAGCCTTTTTTTCTCCCGTTCCATACAGAAGCTGGGCGAACAAACGATGCTCGC
**2799**	GTTTAGCAAAGAGCAACCCAGAGACATTATTTGCCGACTACCTTGGTGATCTCGCCTTTCACG
**2870**	TGTAAGTTTATACATAGGCGAGTACTCTGTTATGGATGCTTAAACAGGTAG
**2871**	TTAAACTTCAACTTGGTAGCC
**ARB2**	GGCCACGCGTCGACTAGTAC
**ARB6**	GGCCACGCGTCGACTAGTACNNNNNNNNNNACGCC
**MV288**	GTCACCGCGGCTGGCTAGCTTCGAACCGGTTCTAGAGACC
**mariner-a-bis**	CACCGTCATGGTCTTTGTAG

Table S4 : Genes identified in the inactivation screen

**Replication Recombination Repair**		
*VC2322	RecC	RecBCD Double-strand break repair
*VC1886	Mfd helicase	Transcription repair coupling factor
*VC0498	RnaseH	RNA-DNA helicase
*VC1636	Putative ($YejH_{vc}$)	DNA-RNA helicase
*VC0016	GreA-like	putative transcriptional regulator
**Oxidative stress and metabolism related**		
*VCA0169-172	*bat* aerotolerance operon	Oxidative stress, induced by c-di-GMP (1)
*VCA0139	YodA-like protein	Possibly, Acetyl-CoA synthesis.<br>YodA is induced by oxidative stress and high cell density (2,3)
*VC0604	Aconitase	Induced by oxidative stress (4)
VC0123	CyaY frataxin	Possible iron donor (5)
VC2559	CysN GTPase	Induced by oxidative stress (6)
VCA0901	Thioredoxin	Oxidant, promoting disulfide bond formation
VC1659-62	Thioredoxin	Oxidant, promoting disulfide bond formation
VC1516	HyfH	Fe-S binding protein
**Membrane proteins**		
VC2632-3	PilO-PilN	Type IV pilus assembly - Induced by c-di-GMP (7)
VC0917	WecB	Peptidoglycan synthesis
*VC0820	TagA	ToxR activated protein. Induced by c-di-GMP (1)
VC0135	PldB	Membrane phospholipid turnover
*VC2635	PBP1 (MrcA)	Peptidoglycan synthesis (8)
VC0950	PBP2 (MrdA)	Peptidoglycan synthesis
**Transporters**		
VCA0179	NupC permease	Repressed by quorum sensing (9)
VCA0834	CzcD	Heavy metal transporter (10)
VCA1012-1013	YjiG-YjiH	Putative iron transporter
VCA0112	Putative type 6 secretion	Membrane modification
VC0609	Iron transporter	Possibly, changes in intracellular iron concentrations
VC2262	GlnD	Iron uptake (11)
VCA0638	MdtC	Multi-drug efflux
VC0337	EamA	Putative transporter
**Two-component systems**		(MCP: methyl accepting chemotaxis protein)
VCA0906	Trg	chemotaxis and flagellar assembly MotAB activation
VC0282	MCP	Signal transduction?
VCA0851	YpdB	2 component histidine kinase
VC0512	MCP	Aerotactic response
VC1831	ArcB	Part of phosphotransfer network regulating RpoS degradation (12)
**Motility**		
VC0893	MotB	Motility
*VC1761-70	MotB-like	Possibly, motility
**Transcriptional regulators**		
*VC0665	VpsR	binds to c-di-GMP - antagonizes HapR (13)
VCA1020	HdfR	activates fimbriae, motility
VC0396	CsgD	LuxR family transcriptional regulator
VCA0094	YtfH	MarR family transcriptional regulator
**GGDEF**		
VC0703	MbaA	c-di-GMP diesterase c-di-GMP concentration increase, induces *hapR*

		expression (14,15)
*VC1216	HemT - YegE	Binds oxygen and iron(16), adjust c-di-GMP concentration for motility control (17) induced by c-di-GMP (1)
VC0398	CsrD	target mRNAs to RNaseE, induce CsgD
Other		
*VCA0762-3	YieMN	moxR-like ATPase and its repressor (18)
VCA0422	YefM	(phd) Antitoxin
VC2030	RNase E	mRNA degradation

*: gene for which deletion mutants were reconstructed and verified in wild type *V. cholerae* N16961 *hapR+* strain. The other mutants were not verified.

Supplementary References

1. Beyhan, S., Tischler, A.D., Camilli, A. and Yildiz, F.H. (2006) Transcriptome and phenotypic responses of Vibrio cholerae to increased cyclic di-GMP level. *J Bacteriol*, **188**, 3600-3613.
2. David, G., Blondeau, K., Schiltz, M., Penel, S. and Lewit-Bentley, A. (2003) YodA from Escherichia coli is a metal-binding, lipocalin-like protein. *J Biol Chem*, **278**, 43728-43735.
3. Puskarova, A., Ferianc, P., Kormanec, J., Homerova, D., Farewell, A. and Nystrom, T. (2002) Regulation of yodA encoding a novel cadmium-induced protein in Escherichia coli. *Microbiology*, **148**, 3801-3811.
4. Chen, X.J., Wang, X. and Butow, R.A. (2007) Yeast aconitase binds and provides metabolically coupled protection to mitochondrial DNA. *Proc Natl Acad Sci U S A*, **104**, 13738-13743.
5. Albrecht, A.G., Landmann, H., Nette, D., Burghaus, O., Peuckert, F., Seubert, A., Miethke, M. and Marahiel, M.A. (2011) The frataxin homologue Fra plays a key role in intracellular iron channeling in Bacillus subtilis. *Chembiochem*, **12**, 2052-2061.
6. Pinto, R., Tang, Q.X., Britton, W.J., Leyh, T.S. and Triccas, J.A. (2004) The Mycobacterium tuberculosis cysD and cysNC genes form a stress-induced operon that encodes a tri-functional sulfate-activating complex. *Microbiology*, **150**, 1681-1686.
7. Karuppiah, V. and Derrick, J.P. (2011) Structure of the PilM-PilN inner membrane type IV pilus biogenesis complex from Thermus thermophilus. *J Biol Chem*, **286**, 24434-24442.
8. Laubacher, M.E. and Ades, S.E. (2008) The Rcs phosphorelay is a cell envelope stress response activated by peptidoglycan stress and contributes to intrinsic antibiotic resistance. *J Bacteriol*, **190**, 2065-2074.
9. Ren, D., Bedzyk, L.A., Ye, R.W., Thomas, S.M. and Wood, T.K. (2004) Stationary-phase quorum-sensing signals affect autoinducer-2 and gene expression in Escherichia coli. *Appl Environ Microbiol*, **70**, 2038-2043.
10. Lopez, G., Latorre, M., Reyes-Jara, A., Cambiazo, V. and Gonzalez, M. (2012) Transcriptomic response of Enterococcus faecalis to iron excess. *Biometals*, **25**, 737-747.
11. Graf, J. and Ruby, E.G. (2000) Novel effects of a transposon insertion in the Vibrio fischeri glnD gene: defects in iron uptake and symbiotic persistence in addition to nitrogen utilization. *Mol Microbiol*, **37**, 168-179.

12. Mika, F. and Hengge, R. (2005) A two-component phosphotransfer network involving ArcB, ArcA, and RssB coordinates synthesis and proteolysis of sigmaS (RpoS) in E. coli. *Genes Dev*, **19**, 2770-2781.
13. Yildiz, F.H., Liu, X.S., Heydorn, A. and Schoolnik, G.K. (2004) Molecular analysis of rugosity in a Vibrio cholerae O1 El Tor phase variant. *Mol Microbiol*, **53**, 497-515.
14. Lim, B., Beyhan, S. and Yildiz, F.H. (2007) Regulation of Vibrio polysaccharide synthesis and virulence factor production by CdgC, a GGDEF-EAL domain protein, in Vibrio cholerae. *J Bacteriol*, **189**, 717-729.
15. Lim, B., Beyhan, S., Meir, J. and Yildiz, F.H. (2006) Cyclic-diGMP signal transduction systems in Vibrio cholerae: modulation of rugosity and biofilm formation. *Mol Microbiol*, **60**, 331-348.
16. French, C.E., Bell, J.M. and Ward, F.B. (2008) Diversity and distribution of hemerythrin-like proteins in prokaryotes. *FEMS Microbiol Lett*, **279**, 131-145.
17. Pesavento, C., Becker, G., Sommerfeldt, N., Possling, A., Tschowri, N., Mehlis, A. and Hengge, R. (2008) Inverse regulatory coordination of motility and curli-mediated adhesion in Escherichia coli. *Genes Dev*, **22**, 2434-2446.
18. Iyer, L.M., Leipe, D.D., Koonin, E.V. and Aravind, L. (2004) Evolutionary history and higher order classification of AAA+ ATPases. *J Struct Biol*, **146**, 11-31.

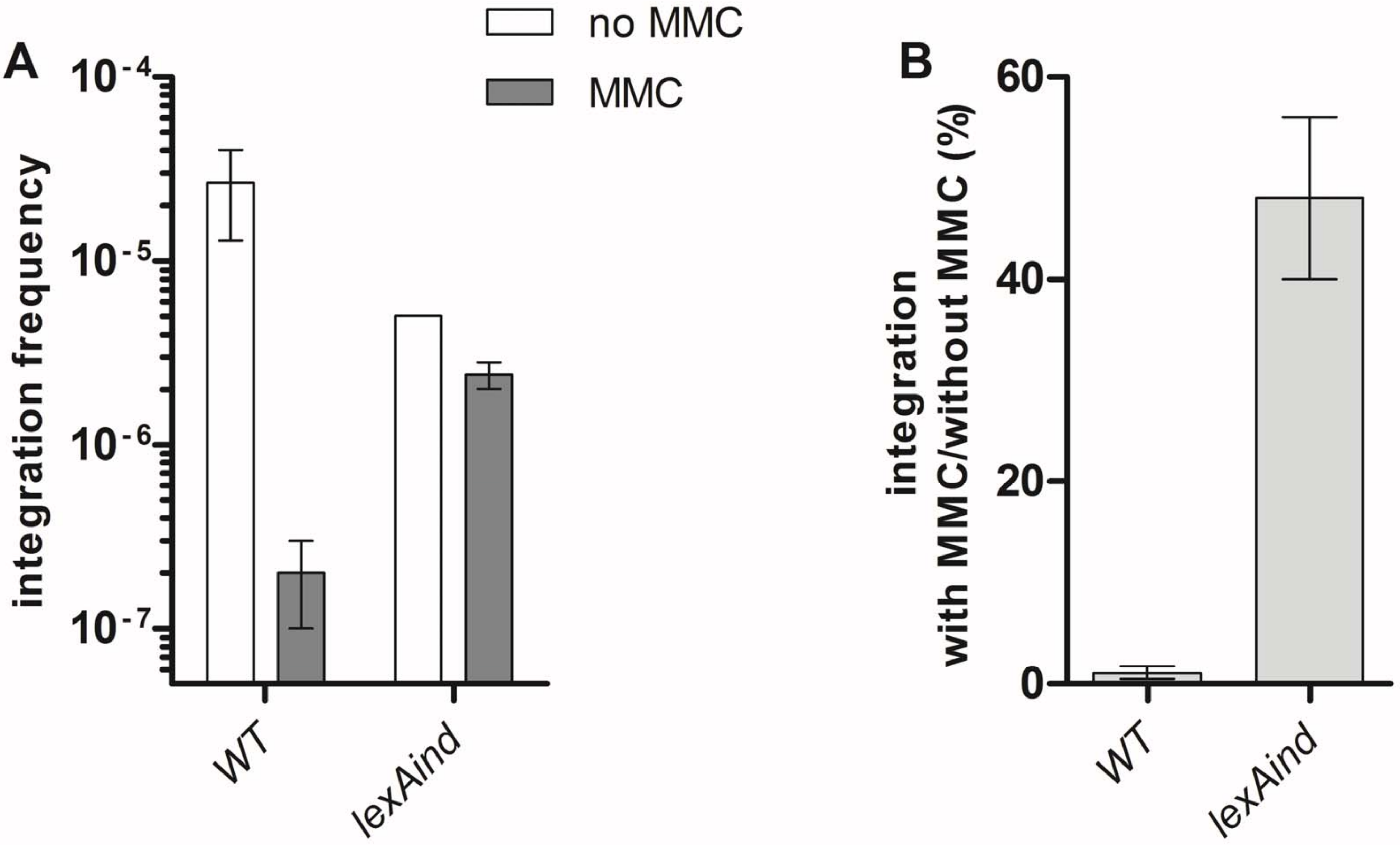
no MMC
MMC
A
integration frequency
10^-4
10^-5
10^-6
10^-7
WT
lexAind
B
integration
with MMC/without MMC (%)
60
40
20
0
WT
lexAind

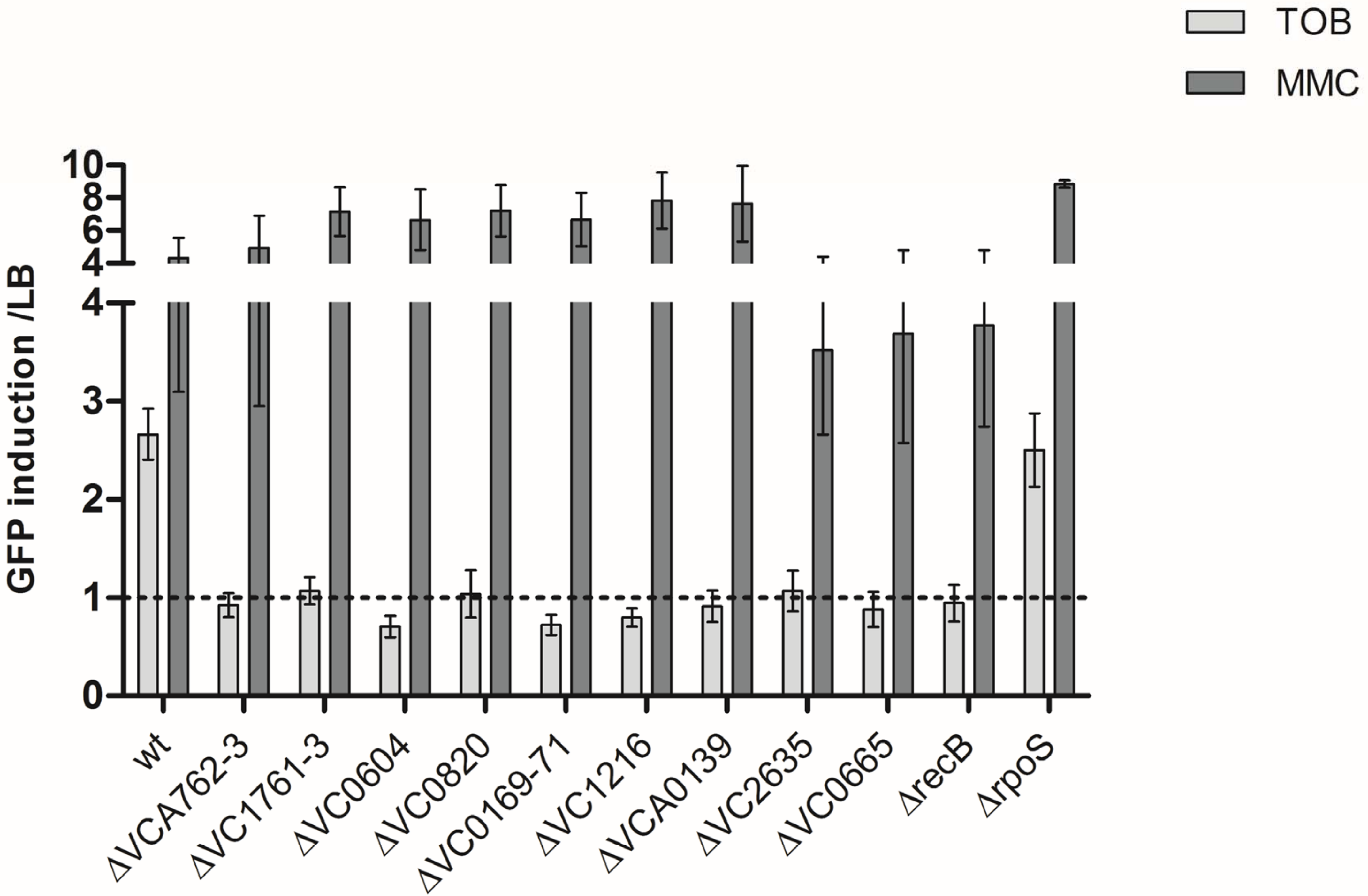

TOB
MMC
GFP induction /LB
10
8
6
4
4
3
2
1
0
wt
ΔVCA762-3
ΔVC1761-3
ΔVC0604
ΔVC0820
ΔVC0169-71
ΔVC1216
ΔVCA0139
ΔVC2635
ΔVC0665
ΔrecB
ΔrpoS

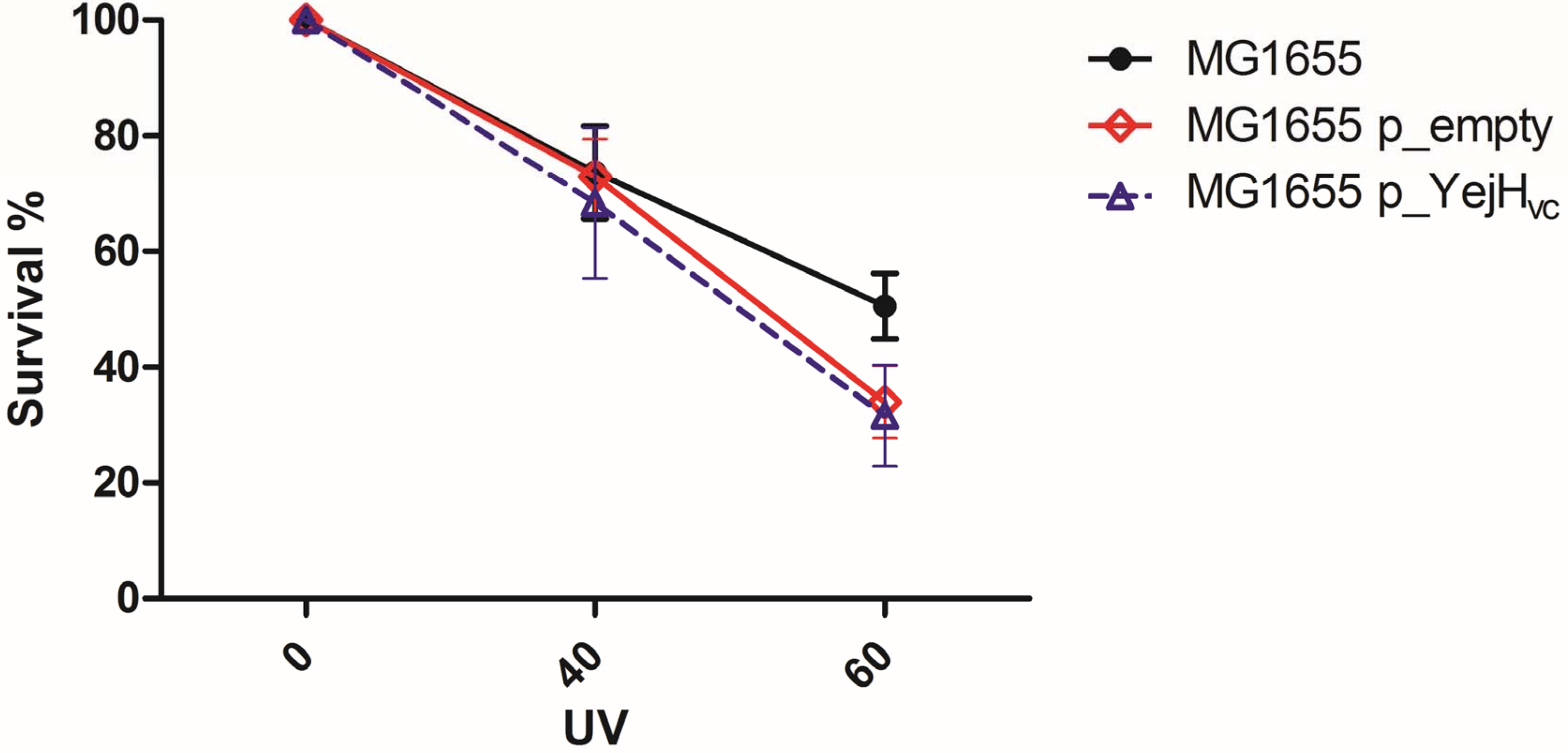

Survival %
100
80
60
40
20
0
0
40
60
UV
MG1655
MG1655 p_empty
MG1655 p_YejH$_{VC}$
